# Efficacy of continuous positive airway pressure on NT-pro-BNP in obstructive sleep apnea patients: a meta-analysis

**DOI:** 10.1186/s12890-023-02539-9

**Published:** 2023-07-14

**Authors:** Qinqin Wu, Xiaojun Ma, Yanyan Wang, Jianfeng Jin, Jia Li, Shuming Guo

**Affiliations:** 1 Department of Pulmonary and Critical Care Medicine, Linfen Central Hospital, Linfen, Shanxi China; 2 Linfen Central Hospital, Linfen, Shanxi China

**Keywords:** N-terminal probrain natriuretic peptide, Obstructive sleep apnea–hypopnea syndrome, Continuous positive airway pressure, Meta-analysis

## Abstract

**Background:**

N-terminal probrain natriuretic peptide (NT-pro-BNP) and BNP are well-known markers for the diagnosis and prognostic of heart failure. Until now, it was not clear whether BNP levels are influenced by events occurring within Obstructive sleep apnea–hypopnea syndrome (OSAHS) with continuous positive airway pressure (CPAP).

**Methods:**

A thorough search in PubMed, EMBASE, Google Scholar, and Web of Science databases up to October 24, 2022, and a meta-analysis aimed to explore further accurate estimates of the effects of BNP on OSAHS after CPAP treatment to assess the strength of the evidence.

**Results:**

The forest plot outcome indicated that CPAP therapy did not change the BNP level in patients with OSAHS, with a weighted mean difference (WMD) of -0.47 (95% CI: -1.67 to 2.62; *P* = 0.53] based on the random effect model because of high significant heterogeneity (*I*^*2*^ = 80%) among the studies. Subgroup analysis also explored the changes in BNP levels in patients with OSAHS. Begg’s test (*P* = 0.835) and Egger’s test (*P* = 0.245) suggested significant negative publication bias.

**Conclusion:**

Our meta-analysis suggests that CPAP therapy does not change the BNP level in patients with OSAHS; therefore, it is not accurate to use BNP level as an index to evaluate heart function in patients with OSAHS, but more related research should be conducted.

## Introduction

Obstructive sleep apnea–hypopnea syndrome (OSAHS) is characterized by repetitive events of partial or whole upper airway obstruction during sleep, resulting in chronic intermittent hypoxemia, excessive daytime sleepiness, irregular snoring at night, and increased nocturia, which are recognized as new controllable risk factors for both cerebrovascular and cardiovascular disease [1, 2]. A previous study reported that 3–9% of female and 10–17% of male have an AHI ≥ 15/h and are present in nearly one billion people between 30 and 69 years of age global [3, 4]. Repeated hypoxic events and concomitant sleep disruption induce ventilatory instability, oxidative stress, inflammation, and disrupts vascular function [5]. Oxidative stress is a key element in the etiology of cardiovascular disease (CVD) [6]. Dietary approaches affect CVD [7–9]. The gold-standard treatment of choice for OSAHS is a device that bring continuous pressure on the upper airway, called continuous positive airway pressure (CPAP), which can correct intermittent hypoxemia, reduces vascular endothelial dysfunction and decreases ventilatory responsiveness to hypoxia [10]. Moreover, CPAP is also a regular ancillary condition, involving intermittent hypercapnia, sleep fragmentation, repetitive increases in negative intrathoracic pressure, sympathetic nerve activity surge, and blood pressure (BP) [10].

Type B natriuretic peptide (BNP) is released mainly by the cardiac ventricles and as a diuretic and vasodilatory hormone, an indicator of pressure load and volume expansion [11]. When ventricular volume load or pressure load increases, pre-proBNP is first secreted, followed by the formation of proBNP [12]. Under the action of endonucleases, proBNP splits into biologically active BNP that is beneficial for sodium, diuresis, vasodilation, and non biologically active N-terminal natriuretic peptide (NT-pro-BNP) [13]. BNP is a peptide composed of 32 amino acid residues, mainly secreted by the heart, and is a member of the diuretic natriuretic peptide family. NT-pro-BNP is a straight chain structure with 76 amino acid fragments that have lost biological activity [14]. The ratio of BNP to NT-pro-BNP production is 1:1, and NT-pro-BNP is mainly cleared by glomerular filtration [15]. When a patient experiences heart failure, as the severity of the condition increases, their cardiac volume load or pressure load will also increase, and the concentration of BNP in the blood will correspondingly increase [12]. Therefore, BNP levels in heart failure patients will increase with the severity of the disease and BNP is a sensitive indicator for diagnosing heart failure and can independently predict the elevation of left ventricular end-diastolic pressure, serving as a better indicator for predicting the condition of heart failure [16]. In the 2004 American expert consensus, the value of NT-pro-BNP was also affirmed and NT-pro-BNP < 300 pg/ml can exclude heart failure, with a negative predictive value of 99% [17]. High NT-pro-BNP levels were associated with heart failure, valvular abnormalities, diabetes mellitus, hypertension, angina pectoris, number of left ventricular hypertrabeculation/non-compaction-affected segments, end-diastolic diameter, and systolic dysfunction [18]. Until now, it has not been clear if BNP level is influenced by events occurring within OSAHS. Patients with moderate/severe OSAS had a larger relative overnight reduction in BNP levels than those with mild/no OSAS [19]. While studies have also reported conflicting data, Møller et al. [20] indicated that there were no differences in BNP levels, regardless of CPAP treatment. As such, this meta-analysis aimed to further explore accurate estimates of the effects of BNP on OSAHS after CPAP treatment to assess the strength of the evidence.

## Methods

### Search strategy

A comprehensive search was conducted in PubMed, EMBASE, Google Scholar, and Web of Science databases up to October 24, 2022, using a set of keywords including “CPAP,” “continuous positive airway pressure,” “BNP,” “brain natriuretic peptide,” “B-type natriuretic peptide,” “natriuretic peptide,” “OSA,” “obstructive sleep apnea–hypopnea syndrome,” “obstructive sleep apnea,” and “OSAHS,” in order to identify relevant original articles. The search strategy was developed by a medical librarian and reviewed by another medical librarian based on PRESS criteria prior to the actual search. Only human studies published in English were included in the analysis. When applicable, chosen study arms or patient subgroups within a study were analyzed. This meta-analysis was conducted in accordance with the PRISMA guidelines for systematic reviews and meta-analyses.

### Study selection

The eligibility criteria were predefined, and two investigators thoroughly searched and screened all titles and abstracts. Any publication that met the eligibility criteria during this phase was retrieved, and the same investigators determined their eligibility based on the relevant full-text publication(s). Relevant systematic reviews were identified during this process and used to cross-check the search strategy for any missed publications. A third reviewer was available to mediate in case of disagreements between the investigators, and the aim was to achieve a comprehensive evaluation of all eligible studies. Furthermore, additional related references cited in the retrieved research were also evaluated.

### Inclusion and exclusion standard

There are no restrictions on the scope of this study. All OSAHS patients underwent CPAP treatment and had their plasma BNP levels measured either before or after the treatment. The study only includes human subjects, is published in English, and provides detailed raw data. Patients with potential comorbidities that could affect BNP levels, such as renal disease and heart failure were not included. The study excludes repeated studies, letters, case reports, abstracts, and comments. The author's name, publication year, sample size, country, CPAP duration, AHI, BMI, and age were collected from the final studies that were included. To assess the quality of each study, the Newcastle–Ottawa Scale (NOS) was used, which evaluates three quality parameters: selection, comparability, and outcomes. The scale has eight specific items and assigns a score ranging from 0 to 9. Studies with a score below 5 are considered to be at high risk of bias.

### Statistical analysis

The meta-analysis used Review Manager software version 5.3 and employed the Newcastle–Ottawa scale to assess each study's quality. Heterogeneity was assessed utilizing the *I*^*2*^ index value, where *I*^*2*^ values of 75–100%, 50–75%, 25–50%, and less than 25% were deemed high, medium, low heterogeneity levels, and homogeneous, respectively. If the *I*^*2*^ value was above 50%, the random effects model (REM) was adopted, while the fixed effects model (FEM) was used if it was less than 50%. For every study, the weighted mean difference (WMD) was determined for each BNP level. Repeated sensitivity analyses were conducted by excluding different individual studies to evaluate their impact. When continuous variables were not available, a conversion of the standard deviation and mean from the median, sample size, and interquartile range (IQR) was performed, according to the Hozo et al. [21] method.

## Results

### Article features

**Fig. 1:**
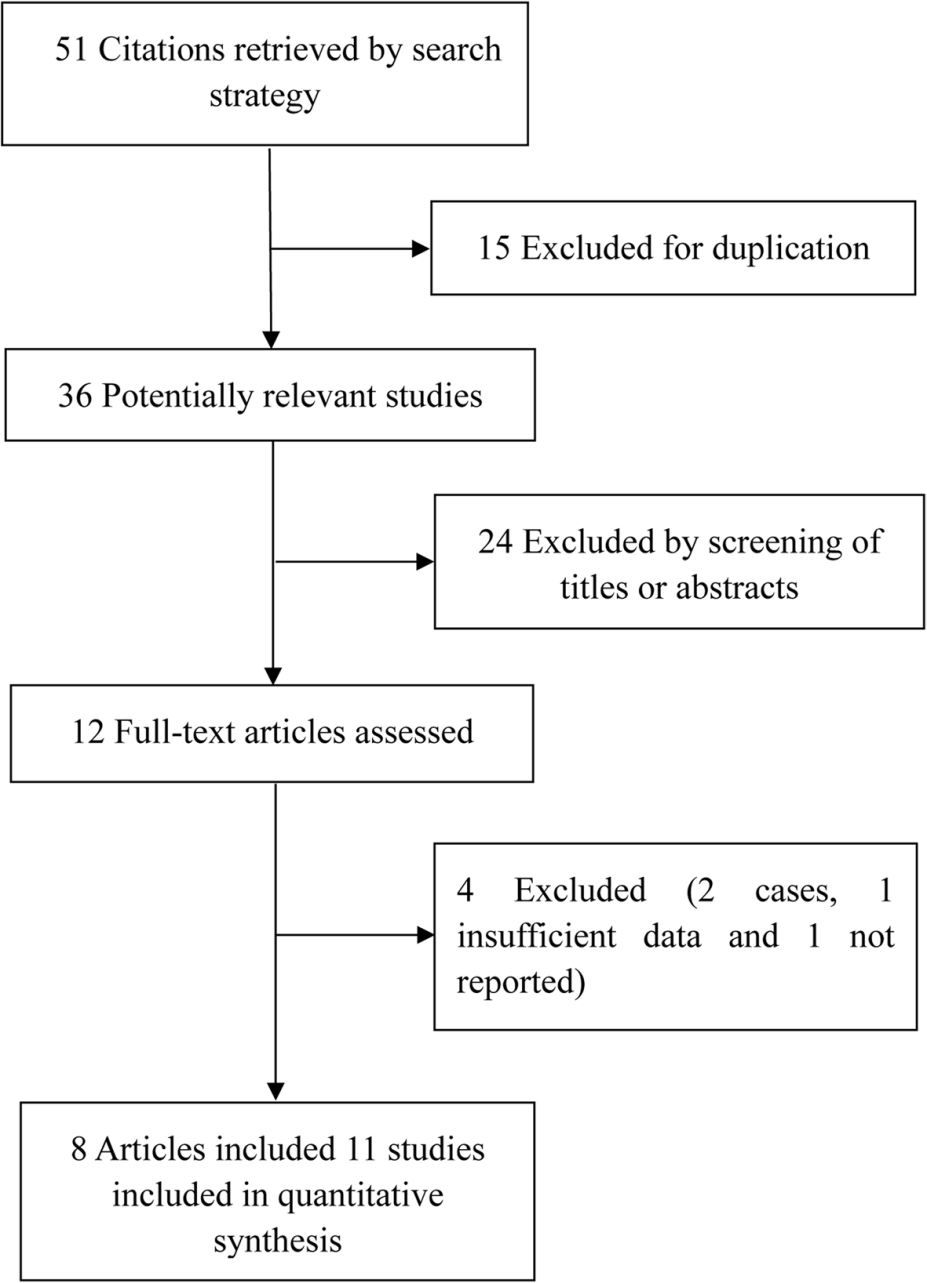
Selection process for studies included in the meta-analysis

A total of 51 relevant articles were identified, but repeated removal of duplicates reduced the number of studies. Thirty-six studies were still screened, and reading the titles and abstracts of these thirty-six studies led us to exclude twenty-four articles that did not meet the inclusion criterion. Four articles were excluded after browsing the full text of the remaining 12 studies and the final meta-analysis included eight articles [20, 22–28]. Figure 1 illustrates the literature retrieval procedure, and Table 1 presents the study details. One study [22] included all patients with OSAHS (Group A), AHI < 30 h (Group B), and AHI ≥ 30 h (Group C). Another study [23] followed up with CPAP therapy for 3 months (Group A) and 12 months (Group B). Five studies [22–26] were self-controlled, and two studies [24, 26] were conducted in Asia. Additionally, one study [24] explored brain natriuretic peptide (BNP) levels in children and adolescents. In Chang et al’s [25] study, the CPAP pressure delivery on sham CPAP was set at 0.5 cmH_2_O and the treatment pressure was set to the 90th centile pressure calculated by an auto-titration machine that controlled most sleep apnoea. In Randerath et al.’s [23] study, the CPAP set by the physician according to the requirements of the patient within the range of 4–30 cm cmH_2_O. All articles scored 6 or above on the NOS, indicating high quality.

**Table 1:** Description of included studies

Study	Country	N (Pre/Post CPAP)	Assay	Age (Pre/Post CPAP)	CPAP time/CPAP set	NOS	AHI (Pre/Post CPAP)	BMI (Pre/Post CPAP)
Muller et al., 2003 [20]	Germany	10/25	commercial chemiluminescent immunoassay	49/49	30 days/NA	6	40.9 ± 23.9/42.2 ± 24.4	28.2 ± 3.8/30.8 ± 5.1
Li et al., 2014 [24]	China	15	chemiluminescent immunoassay	10.8 (6.6–13.3)/ 11.1 (7.4–14.8)	12 weeks/NA	7	19.5 (9.6–33.3)/0.8 (0.5–1.5)	1.20(− 0.18–1.91)/1.40(0.17–1.83)
Chang et al., 2017 [25]	Australia	28	immunoassay	48 ± 12	8-week/sham CPAP: 0.5 cmH_2_O, treatment pressure: 90th centile pressure	7	40.26 ± 27.4/6.97 ± 11.3	31.95 ± 4.22
Craig et al., 2015 [27]	UK	158/158	immunoassay	57.2 ± 6.5/57.6 ± 7.2	6-month/given on an individual basis	8	NA	31.8 ± 4.9/31.9 ± 5.5
Maeder et al., 2009 A [22]	Switzerland	40	chemiluminescent immunoassay	50 ± 9	7.9-month/NA	7	37 (20–65)	30.3 ± 4.5
Maeder et al., 2009 B [22]	Switzerland	16	chemiluminescent immunoassay	49 ± 8	8-month/NA	7	17 (11–26)	30.6 ± 5.6/30.1 ± 5.1
Maeder et al., 2009 C [22]	Switzerland	24	chemiluminescent immunoassay	51 ± 10	7-month/NA	7	54 (40–74)	30.0 ± 3.7/30.0 ± 3.7
Miyazaki et al., 2015 [26]	Japan	32	immunoassay	NA	3–6 month/NA	8	45.3 ± 13.6/2.5 ± 3.7	NA
Randerath et al., 2012 A [23]	Germany	36/34	immunoassay	NA	3-month/4–30 cmH_2_O	6	10.8 ± 11.3/16.5 ± 17.3	NA
Randerath et al., 2012 B [23]	Germany	36/34	immunoassay	NA	12-month/4–30 cmH_2_O	6	11.1 ± 11.6/17 ± 17.9	NA
Strehmel et al., 2016 [28]	Germany	21	immunoassay	61 ± 11	NA/NA	7	53 ± 21	35 ± 7

*NA* not given, Maeder et al.: Group A means all, Group b AHI < 30 h and Group c means AHI ≥ 30 h; Randerath et al.: Group A means follow up 3 months and Group B means follow up 12 months

### Pooled analysis

**Fig. 2:**
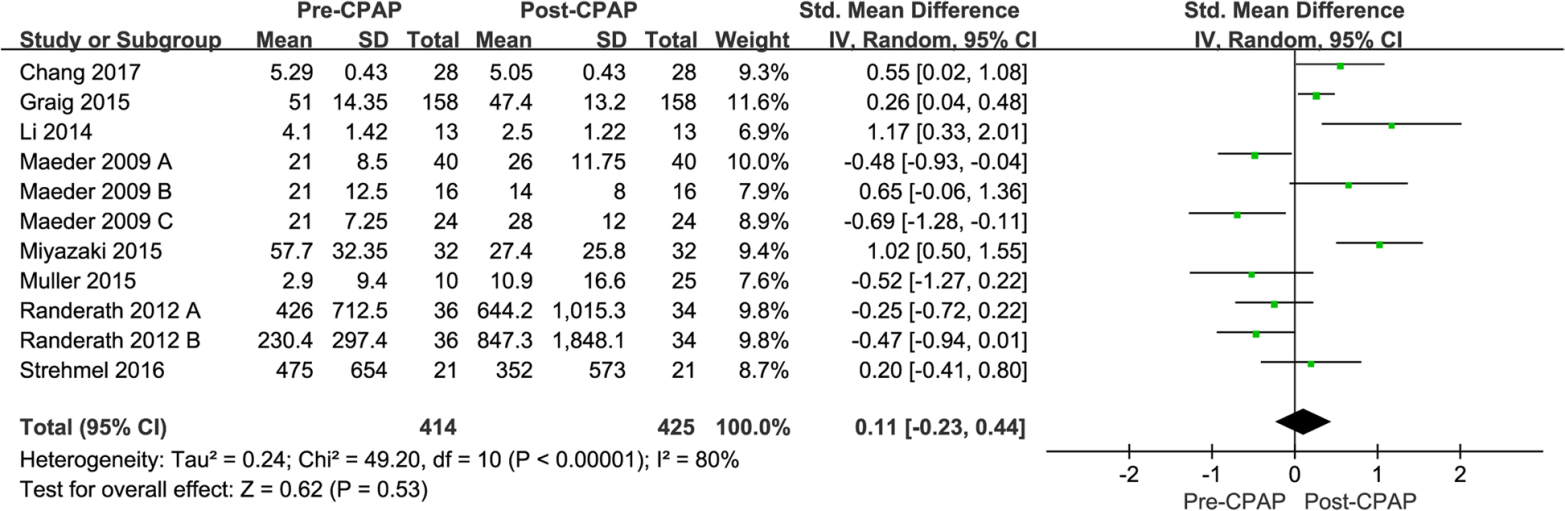
The forest plot outcome indicated that the CPAP therapy will not change the BNP level in OSAHS patients

According to the forest plot result, the use of CPAP therapy did not result in any significant change in the BNP level of OSAHS patients. The WMD was -0.47 (95% CI: -1.67, 2.62; *P* = 0.53) based on the REM, and this could be attributed to the significant variability (*I*^*2*^ = 80%) among the studies, as shown in Fig. 2.

### Subgroup analysis

Subgroup analysis also explored the changes in BNP levels in patients with OSAHS. In the region subgroup, publication year subgroup and self-control study were also non-significant; the full information is shown in Table 2. In Asia subgroup, with an WMD of 14.59 (95% CI: 6.17 to 34.49; *P* < 0.001) based on the FEM since as a high significant heterogeneity (*I*^*2*^ = 34%) among the studies. In Europe subgroup, with an WMD of 7.82 (95% CI: 2.94 to 20.75; *P* < 0.001) based on the FEM since as a high significant heterogeneity (*I*^*2*^ = 0) among the studies. In publication year: before 2000 subgroup, with an WMD of 6.47 (95% CI: 2.76 to 15.21; *P* < 0.001) based on the FEM since as a high significant heterogeneity (*I*^*2*^ = 0) among the studies. In publication year: after 2000 subgroup, with an WMD of 8.85 (95% CI: 4.63 to 16.94; *P* < 0.001) based on the FEM since as a high significant heterogeneity (*I*^*2*^ = 50) among the studies. In NOS < 7 subgroup, with an WMD of 9.18 (95% CI: 4.31 to 19.53; *P* < 0.001) based on the FEM since as a high significant heterogeneity (*I*^*2*^ = 8) among the studies. In NOS = 7 subgroup, with an WMD of 6.83 (95% CI: 3.37 to 13.85; *P* < 0.001) based on the REM since as a high significant heterogeneity (*I*^*2*^ = 56) among the studies. Finally, Begg’s test (*P* = 0.835) and Egger’s test (*P* = 0.245) suggested significant negative publication bias.

**Table 2:** Results of subgroup analysis among included studies

Subgroup	Studies included (N)	Heterogeneity		Pooled WMD
		*I*^*2*^ (%)	*P* value	
Self-control study	5	83	0.25	0.32 (-0.22–0.86)
Asia	2	86	0.11	0.6 (-0.14–1.35)
Publication year: non 2015	2	0	0.05	0.4 (-0.01–0.8)
Country: Germany	3	11	0.08	-0.26 (-0.56–0.03)
Country: Europe	5	70	0.58	-0.11 (-0.49–0.27)

## Discussion

Our meta-analysis recommends that CPAP therapy does not change the BNP level in patients with OSAHS. Our study also showed that there was high heterogeneity. Moreover, the sensitivity analysis showed that the overall results remained unchanged when any single study was excluded or REM was converted to FEM.

A previous study indicated that serum adiponectin levels were positively associated with serum NT-pro-BNP levels, while adiponectin levels were significantly increased in heart failure patients aged > 70 years old [29]. Kita et al. [30] found that during the sleep period (02:00–06:00 h), BNP levels increased during the night before CPAP treatment and were reduced by effective CPAP, and BNP levels were significantly related to BP elevation and apnea duration. While studies have also reported conflicting data, Li et al. [24] suggested that BNP levels did not differ between patients in both non-obese and obese OSAHS groups and that CPAP intervention could not change BNP levels. Moreover, Cifçi et al. [31] not detected any significant differences between the severity of OSAHS and serum pro-BNP levels. A large number of studies have shown that CPAP intervention in OSAHS can reduce cardiac damage and injury and improve cardiac function [32]. Therefore, it is logical to hypothesize that CPAP also leads to a reduction in BNP level. However, this is not a consistent finding in research to date. One study found a distinct rise in BNP in the latter half of sleep [20], while another suggested a contradictory fall in BNP levels after sleep relative to before sleep [22]. But, given the limited evidence that CPAP may help lower cardiac biomarkers in OSAHS patients diagnosed with coronary artery disease, it is essential to conduct randomized controlled trials specifically for these high-risk groups in the future. The potential mechanism that can explain the change in the BNP level in patients with OSAHS is that BNP was positively related to high blood pressure and CPAP confirmed that it can reduce blood pressure [20, 33] and this may be interpreted as a reduction in BNP levels [25]. Meanwhile, CPAP can alleviate nocturnal hypoxemia, as decreased oxygen saturation stimulates BNP secretion via enhanced transcriptional activity of hypoxia-inducible factor-1 [34]. However, even without cardiac dysfunction, chronic hypoxia alone is sufficient to increase BNP levels, which may counteract pulmonary vasoconstriction by hemostasis and automatic regulation of vascular tension [35]. So date, no meta-analysis has explored BNP level changes after CPAP treatment in patients with OSAHS to strengthen the evidence. Therefore, our meta-analysis found that CPAP therapy does not change BNP levels in patients with OSAHS, which may contribute to better clinical management of OSAHS patients.

As a quantitative marker of heart failure, BNP reflects left ventricular systolic, left ventricular diastolic, right ventricular, and valve dysfunction [36]. BNP is mainly used for emergency diagnosis [37]. As OSAHS is a relatively non-acute disease [38], this may explain why BNP changes are not obvious in patients with OSAHS. In this study, we also found a negative result in the subgroup analysis, which means that changes in clinical characteristics will not affect the changes in BNP levels in patients with OSAHS, but more related research should be conducted. However, due to the lack of data, we could not investigate the characteristics of BMI and AHI in the impact of BNP in OSAHS patients [39], and additional correlated studies on the association between BMI and AHI stratification should be conducted. Importantly, the respiratory setting of the cpap may influence the level of BNP. Dividing CPAP into two components, inspiratory positive airway pressure (IPAP) and expiratory positive airway pressure (EPAP), is a conceptual approach that can be taken. IPAP helps reduce respiratory effort by providing additional inspiratory pressure and decreasing inspiratory muscle work. EPAP counters the inspiratory threshold caused by auto-PEEP and increases intraluminal pressure by keeping the airway open, thereby preventing dynamic airway collapse [40]. Additionally, EPAP may engage expiratory muscles, which assist in maintaining end-expiratory lung volume and help reduce inspiratory effort [41]. The clinical relevance of separating the effects of EPAP from those of IPAP lies in the widespread use of bilevel positive airway pressure as a treatment for patients with airflow obstruction. The level of PEEP, IPAP and Fio2 may influence both the mean airway pressure and the oxygenation trigger, in our study, we can not perform subgroup analysis on this point as the data is insufficient.

To the best of our knowledge, this study is the first attempt to prove whether OSAHS is related to BNP and CPAP treatment through a meta-analysis. At the same time, our research also has limitations. First, an AHI > 5 indicates OSA and is categorized as mild, moderate, or severe based on increasing AHI values and research has revealed that certain demographic and anthropometric factors, such as male gender, higher age and increased body weight, pose a greater risk for developing OSA. Improving compliance with OSA is an important aspect of patient management. Research has shown that only 50% to 60% of patients who achieve medical insurance compliance after 3 months of CPAP treatment, and 40% to 50% of patients with longer term compliance [42]. While due to insufficient data, we have no information about BMI, AHI, age, sex, CPAP time, etc. Second, owing to the different susceptibility and measurement methods of BNP, the results may be biased. Finally, primary prevention requires a large number of subjects and a long follow-up period, which may lead to information deviation and affect the accuracy of the results. However, our study had strengths such as the fact that CPAP therapy did not change the BNP level in OSAHS patients, especially in subgroups. A research was conducted on patients with no confirmed cardiovascular disease who underwent coronary angiography, which revealed that a rise of 50 pg/ml in BNP was linked with a higher possibility of coronary artery stenosis (OR 2.367) [43]. A decrease in BNP levels may be possible as CPAP therapy has demonstrated its effectiveness in reducing blood pressure. Biomarkers like BNP that have both diagnostic and predictive roles could be beneficial in primary prevention clinical studies, which typically involve a significant number of subjects and lengthy follow-up periods.

## Conclusion

Our meta-analysis suggests that CPAP therapy will not change the BNP level in patients with OSAHS; therefore, it is not accurate to use BNP level as an index to evaluate heart function in patients with OSAHS, but more related research should be conducted. In addition, in the region subgroup, publication year subgroup and self-control study subgroup analysis was also indicated that CPAP therapy will not change the BNP level in patients with OSAHS.

## Data Availability

All data generated or analysed during this study are included in this published article.

## Abbreviations

NT-pro-BNP: N-terminal probrain natriuretic peptide
CPAP: Continuous positive airway pressure
OSAHS: Obstructive sleep apnea–hypopnea syndrome
